# Protein sequence and structure alignments within one framework

**DOI:** 10.1186/1748-7188-3-4

**Published:** 2008-04-01

**Authors:** Gundolf Schenk, Thomas Margraf, Andrew E Torda

**Affiliations:** 1 Centre for Bioinformatics, University of Hamburg, Bundesstr. 43, D-20146 Hamburg, Germany

## Abstract

**Background:**

Protein structure alignments are usually based on very different techniques to sequence alignments. We propose a method which treats sequence, structure and even combined sequence + structure in a single framework. Using a probabilistic approach, we calculate a similarity measure which can be applied to fragments containing only protein sequence, structure or both simultaneously.

**Results:**

Proof-of-concept results are given for the different problems. For sequence alignments, the methodology is no better than conventional methods. For structure alignments, the techniques are very fast, reliable and tolerant of a range of alignment parameters. Combined sequence and structure alignments may provide a more reliable alignment for pairs of proteins where pure structural alignments can be misled by repetitive elements or apparent symmetries.

**Conclusion:**

The probabilistic framework has an elegance in principle, merging sequence and structure descriptors into a single framework. It has a practical use in fast structural alignments and a potential use in finding those examples where sequence and structural similarities apparently disagree.

## Background

Protein sequence alignments usually rely on a substitution matrix. This reflects an evolutionary model and the probability that one type of residue has mutated to another [1,2]. Protein structures can also be aligned, but using a very different set of heuristics. Here, we propose a single framework which estimates the similarity of small protein fragments and can be applied to sequence, structure or both simultaneously. The cost is that one has to discard the evolutionary model and replace it with one based purely on descriptive statistics. The benefit is that after the initial approximations, one has a rather rigorous measure of the similarity of pieces of proteins.

Just considering sequences, there is already a history working with different sized fragments. Firstly, one can think of conventional sequence alignment as working with fragments of length *k*, where *k *= 1. There is plenty of data to estimate the log-odds probabilities of 20 × 21/2 = 210 possible mutations [2]. Since sites in a protein are not independent, one could try to build a substitution matrix for *k *= 2 (dipeptides) [3,4]. Unfortunately, there is simply not enough data to estimate all of the 400 × 401/2 = 80 200 mutation rates [4,5]. The direct parameter estimation requires that all mutations be observed and, for reliable statistics, frequently observed.

Proteins can also be aligned on the basis of their structures, but there is no single popular methodology. Structure reflects the arrangement of residues in space and is not a property of a single residue, so fragments with *k *= 1 will never be a good way to represent structural properties. Furthermore, of the mass of structural alignment methods [6-34], hardly any explicitly try to estimate log-odds probabilities as a similarity measure [35].

If one is willing to forget the evolutionary model, it should be possible to statistically measure fragment similarity, but based on what is observed, rather than requiring that everything possible be observed. Furthermore, one should be able to work with larger fragment lengths. A fragment could be characterised by some vector of properties and the similarity of two such vectors would measure the similarity of the fragments.

This has been done using physical or chemical properties which seem reasonable to a chemist [16,28], but we have aimed for a more objective statistical approach. In this work, long vectors are created, but they come from a probabilistic classification procedure. With *N*_*c *_classes, a fragment has a vector of probabilities that it is in class 1, 2, ...*N*_*c*_. Given this vector for two such fragments, one can then ask, what is the probability that two fragments are in the same class ? Regardless of which class this is, similar fragments will have similar vectors of probabilities. The classification may not be perfect, so some fragments may have a non-zero probability of being in several classes. Even if one cannot say which class the fragments are in, similar fragments will have similar patterns of probabilities. This could be seen by the dot product of class membership probability vectors and is formalised below (eq. 4). In this work, the classification comes from a maximally parsimonious Bayesian classification of fragments. The number of classes is typically of the order of 10^2^, the fragment length *k *= 6 and the amount of training data of the order of 10^6 ^observations.

The classes used here are sets of statistical distributions. These are multinomial Bernoulli distributions for the discrete (sequence) properties, Gaussian for the continuous (structural) properties and appropriate mixture models to combine sequence and structure. For example, one may have a pure structure classification based on *φ *and *ψ *backbone angles. One class within such a classification would have *k *pairs of *φ *and *ψ *distributions (one for each of the *k *residues). Given some observation (fragment), one can can calculate its probability of being in a class by calculating the probability of each *φ*,*ψ *pair within the corresponding distributions that define the class and taking the product of these probabilities.

Exactly the same process can be applied to sequence by using distributions of amino acid probabilities at each of the *k *sites within a class. Instead of Gaussian distributions, one has 20-way probabilities at each site. Different classes will reflect the different probabilities of finding each amino acid at each position. Class membership of a fragment is simply calculated from the product of the probabilities of each amino acid occurring at each site within the class.

Finally, a classification can combine sequence and structure distributions. Class membership of a fragment is just the product of probabilities of finding its sequence (discrete) and structure (continuous) descriptors within some class.

In practice, structure classes were based on bivariate Gaussian distributions in order to account for the strong correlation of *φ*,*ψ *angles within residues.

Describing proteins by fragments is not new, but the philosophy here differs from most literature examples [36-39]. Firstly, the classification is probabilistic. A fragment is never a member of just one class. It may have 0.99 probability of being in one class or it may have partial membership of a few classes. This is particularly important for robustness in comparison problems as described below. Secondly, the clustering does not rely on an explicit measure of cluster similarity or distance. Instead, a model is constructed for the data and the likelihood of the model is optimised, with no need to explicitly consider distances between clusters. Finally, there are almost no preconceptions built into the clustering since we rely on unsupervised learning. If α-helices, β-strands or sequence patterns of hydrophobicity and hydrophilicity are found, it is a consequence of fitting a statistical model, not chemical preconceptions.

## Methods

### Data sets

The training data was a set of protein chains taken from the protein data bank (PDB) [40] such that no two members of the list had more than 50 % sequence identity [41]. After removing all chains with less than 40 residues and the few with unknown sequence, each possible overlapping fragment of length *k *was extracted. Fragments with any bond longer than 2 Å were discarded, leaving a set of just over 1.5 × 10^6 ^fragments of length *k *≤ 6.

A set of protein pairs was used for testing alignments and selected so that there should be some structural similarity, but little sequence similarity. Starting from a list of related pairs of proteins [42-45], a set of 2 902 pairs was selected by requiring the members of the pair have less than 19 % sequence identity, but were superimposable to 3 Å or less over at least 40 residues.

### Classification

For classifications based only on sequence, each of the *k *residues in each class was modelled by a 20-way categorical (multi-way Bernoulli) distribution. For classifications using backbone angles, *φ *and *ψ *of each residue were shifted into the periods of 0 to 2*π *and -*π*/2 to 3*π*/2 respectively and treated as continuous descriptors. To allow for correlations between *φ *and *ψ *angles, they were modelled as bivariate Gaussian distributions of the form

(1) p(θ)=exp⁡(−12(θ−μθ) C−1(θ−μθ)T)((2π)2|C|)12

where **θ **is the two-dimensional vector for a *φ*, *ψ *pair and **μ**_*θ *_is the corresponding vector of means. **C **is the covariance matrix, |**C**| the absolute value of the corresponding determinant and (**θ**-**μ**_**θ**_)^T ^is the transpose of (**θ**-**μ**_**θ**_). Classifications using both sequence and structure used a mixture model with both the discrete and continuous distributions.

Given the distribution types, expectation maximization was used to find the model (parameter set) which maximises the likelihood of the data [46]. One uses an initial guess for the distribution parameters and re-estimates the distribution properties. These estimates are then used to re-calculate the distribution properties and the process iterated until a maximum in terms of likelihood is reached. This is usually a local maximum, so the entire classification process is repeated many times.

Probability calculations were done in wurst [47], but the classifications were constructed using the implementation of Cheeseman and Stutz searching over both the distribution parameters and number of classes [48]. This probabilistic approach leads to some useful results. There is a formal method for estimating the relative probability of a classification. Firstly, one has to be able to calculate a probability *P*(**f**_*i *_∈ *c*_*j*_) that a fragment *i *with its vector of attributes (angles, sequence) **f**_*i *_is a member of class *c*_*j*_. This depends on the product of the probability of seeing each of the *m *attributes in each of the distributions

(2) $$P\left(f_{i}|v_{j}\right)=w_{j}\prod_{m}P\left(f_{i,m}|f_{i}\in c_{j},v_{j,m}\right)$$

where **v**_*j *_is the set of distribution properties describing class *j*. *w*_*j *_is the weight or probability associated with class *j*. The product runs over the *m *attributes and considers the parameters **v**_*j*,*m *_which describe the *m*'th attribute in the *j*'th class. When calculating the probability of a fragment being in a class, eq 2 is applied to all classes and normalised so that the sum of probabilities is one. The class weights, *w*, reflect the importance of a class and are subject to the normalisation $\sum_{j}w_{j}=1$.

There are two more consequences. Firstly, there is a measure for the relative success of a classification. The probability of the database *F *of fragments depends on the probability of seeing all of the contributing fragments and the set *V *of all **v**_*j*,*m*_

(3) $$P\left(F|V\right)=\prod_{i}\left(\sum_{j}\left(w_{j}\prod_{m}P\left(f_{i,m}|f_{i}\in c_{j},v_{jm}\right)\right)\right)$$

and this introduces a strong element of parsimony. Any time new parameters are introduced, one brings in a multiplicative factor less than one. Thus, any time a new class is introduced, the probability of the data set appears to decrease unless the new class is strongly supported by the data. This means the method is not very susceptible to overfitting and there is a tendency to find the minimal number of classes necessary to model the data.

The search over parameters can then be summarised. For a given trial number of classes, distribution parameters were initially chosen randomly, optimized with expectation minimization and the process repeated many times. This was then repeated so as to optimise the number of classes. For a fragment length of *k*, this leads to a number of parameters to optimize as shown in Table 1.

**Table 1:** Parameters optimized during clustering

Classification	*n*_*disc*_	*n*_*cont*_	*n*_*total*_
sequence	20*k*	0	20*k*
structure	0	5*k*	5*k*
sequence + structure	20*k*	5*k*	25*k*

For the three types of classification and for fragments of length *k*, *n*_*disc *_is the number of adjustable parameters associated with discrete distributions, *n*_*cont *_the number of adjustable parameters associated with continuous (multivariate Gaussian) distributions and *n*_*total *_the total number of adjustable parameters.

### Similarity and alignments

Given a classification, it could then be used for the calculation of alignments. If a classification is based on fragments of length *k*, then a protein with *n*_*r *_residues is broken into *n*_*r *_- *k *+ 1 overlapping fragments. The class membership probabilities could then be assigned using eq 2. Given *n*_*c *_classes, a fragment is characterised by an *n*_*c*_-dimensional vector, so a protein can be seen as *n*_*r *_- *k *+ 1 vectors in an *n*_*c*_-dimensional space. Given two such protein fragments *i *and *j*, probably from different proteins, one can calculate a similarity measure *s*_*ij*_

(4) *s*_*ij *_= **p**_*i*_·**p**_*j*_

where **p**_*i *_denotes the vector of class probabilities for fragment *i*. If the two vectors have been normalised to unit vector length, *s*_*ij *_offers a rather rigorous measure of similarity in the range 0 to 1. These s_*ij *_scores can be used as the elements of a similarity matrix suitable for calculating optimal pairwise alignments. The procedure can be applied to probabilities calculated from pure sequence or pure structure or combined sequence and structure. Unlike conventional scoring methods, eq. 4 does not relate to single sites or amino acids in a protein. The vector **p**_*i *_reflects *k *residues. This means that each entry *s*_*ij *_in the score matrix reflects the contribution of *k *overlapping fragments, each of length *k*, so it is sensitive to an environment of 2*k *- 1 residues. All alignments were calculated with wurst [47] using the Gotoh version [49] of the Smith and Waterman [50] algorithm and with parameters optimized as described below.

In order to optimize alignment parameters or measure the quality of alignments, a cost function was used which does not rely on any reference or ideal alignments. Given a pair of proteins "A" and "B" of known structure, they can be aligned by some method such as sequence alignment. From the alignment, a backbone model for "A" can be calculated using the coordinates of "B". The operational definition of alignment quality is a geometric measure for how close the model is to the original coordinates for "A". This can be calculated and averaged over the set of 2 902 protein pairs (described above) and done for both AB and BA pairs. The structural measure used is similar to the *Q*-value common in the folding literature which quantifies how many correct contacts are made within a protein [51,52]. First, one calculates the difference between C^*α *^based distance matrices [53,54], sometimes referred to as the distance matrix error (DME) [55,56]

(5) DMEnat,model=(2Nres(Nres−1)∑i<jNres(rijnat−rijmodel)2)12

where $r_{ij}^{nat}$ is the distance between C^*α*^_*i *_and C^*α*^_*j *_in the native structure and $r_{ij}^{model}$ is the corresponding distance in the model and the summation runs over the *N*_*res *_aligned residues. Next, one defines a threshold, *DME*^*cut *^= 4.0 Å, bearing in mind the typical C^*α *^- C^*α *^distance between adjacent residues is 3.8 Å. Then one discards the elements where the two distance matrices are most different, until *DME*_*nat*,*model *_is less than or equal to *DME*^*cut*^. The remaining fraction of the distance matrix is *f*({**r**^*nat*^},{**r**^*model*^}) where {**r**^*x*^} is the set of C^*α *^coordinate vectors from molecule *x*. In pseudocode, one can describe the process:

   while (DME_*nat*,*model *_>DME^*cut*^) {

      remove largest distance difference from C^*α *^distance matrix

      recalculate *DME*_*nat*,*model*_

      *f*({**r**^*nat*^},{**r**^*model*^}) = fraction of distance difference matrix remaining

   }

To convert this to a penalty function, one notes that *f*({**r**^*nat*^},{**r**^*model*^}) near 1 means structures are nearly identical, but below about 0.5, there is little similarity. This leads to the use of a smooth switching (sigmoidal) function centred at 0.7. The final cost function *C *is then

(6) $$C=-\frac{1}{N_{pair}}\sum_{i=1}^{N_{pair}}{\left(1+e^{b\left(a-f\left({\vec{r}}_{nat_{i}},{\vec{r}}_{model_{i}}\right)\right)}\right)}^{\;-1}$$

where *a *= 0.7 and *b *= 15 (an arbitrary choice for the shape of the sigmoid). The summation ran over all *N*_*pair *_= 2 902 protein pairs.

**Figure 1:**
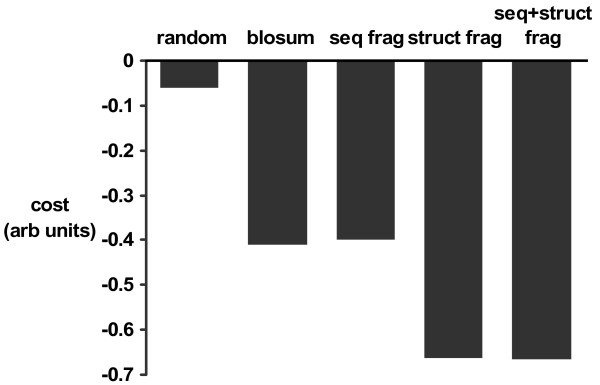
**Alignment quality from different methods**. Each bar shows the value of the cost function (eq. 6) for alignments after optimizing gap penalties and zero-offset added to score matrices. Random: score matrices filled with gaussian distributed random numbers; blosum : a blosum62 matrix, seq frag: fragments from a pure sequence classification, struct frag: fragments from a pure structural classification; seq+struct frag: fragments from a combined sequence and structure classification.

Given this measure of alignment quality, parameters were optimized with a simplex optimizer as previously described [57]. In this work, gap penalties were optimized as well as a constant zero-offset added to each scoring matrix. This is necessary since the scoring procedure provides only positive numbers. This structure-based cost function was used as the measure of alignment quality (Figure 1) with 90 % of the data used for optimizing and 10 % reserved for testing.

The one psi-blast database search referred to below used a profile built with acceptance parameters orders of magnitude more careful than default values [58]. 15 iterations were run, accepting homologues from the non-redundant protein sequence database with an *e*-value < 10^-10^, 10 iterations with the threshold set at 10^-8 ^and 5 iterations with the threshold set at 2 × 10^-5^. This profile was then used as a query against sequences derived from protein data bank structures. For comparison, the default acceptance threshold is *e*-value < 5 × 10^-3^.

## Results

### Classification in general

**Figure 2:**
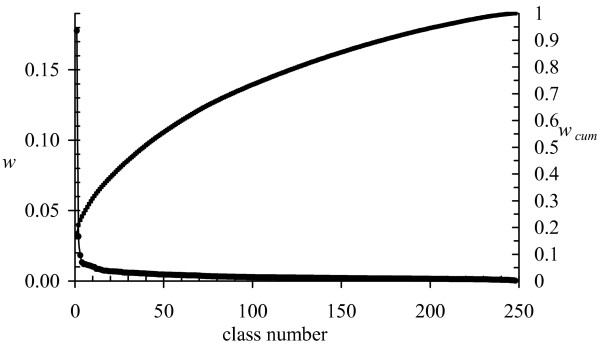
**Number of classes**. The relative weight, *w*, or importance of each class is shown as well as the cumulative probability, *w*_*cum *_accounted for by the classes taken from a pure structure classification with *k *= 6. Lines connecting the points have no meaning, but are used to guide the eye.

Fragment classifications were attempted for pure sequence, pure structure and combined sequence+structure and for fragment lengths up to *k *= 6. Larger values of *k *may be desirable and there may be ample data (1.5 × 10^6 ^data points), but the parameter search space becomes intractably large. One should note that one never finds the optimal classification or even the correct number of classes for any realistic problem. The next point is that it is not always meaningful to simply quote the number of classes. For fragment length *k *= 6 and a pure structural classification, a good classification was found with 248 classes, but this number alone is misleading. One can estimate the importance of each class by summing class membership probabilities over all data points and their partial class memberships. Figure 2 shows the importance of each class after ranking them. The first class accounts for nearly 18 % of the data and 80 % of the observations are accounted for by 125 classes. Although the numbers depend on the kind of classification, this property is clear. There may be a large number of classes, but their importance varies tremendously so the more common classes are well characterized.

Finally, one can see how well a classification reflects the original data. First, the training data was put into bins of 0.4 × 0.4 radians in the *φ *and *ψ *dimensions and treated as a probability distribution which could be compared against probabilities from a classification. The first measure was the Kullback-Leibler divergence, *D*_*KL *_given by

(7) $$D_{KL}=\sum_{i,j}p_{ij}^{H}\ln\frac{p_{ij}^{H}}{p_{ij}^{C}}$$

where the superscript *H *denotes a histogram from the training data, *C *denotes the classification and *p*_*ij *_is the probability for bin *i*,*j*. *D*_*KL *_is zero if two distributions are the same and grows as they differ. Similarly, one can treat the two-dimensional histogram from the training data and probabilities from the classification as vectors and then calculate a dot product. This will equal 1 if the two distributions are the same.

**Figure 3:**
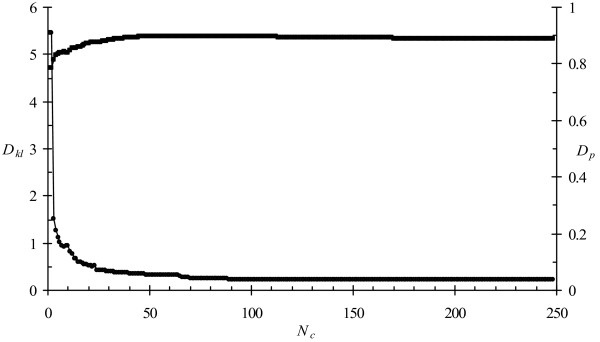
**Quality of classification**. Kullbach-Leibler divergence, *D*_*kl *_(circles) and dot product *D*_*p *_(squares) as a function of *N*_*c*_, the number of classes in a classification. Lines connecting the points have no meaning and are only to guide the eye.

Using the same classification as above, *D*_*KL *_= 0.22, whereas a random distribution gives *D*_*KL *_= 2.01. Labelling the dot product as *D*_*p*_, we find *D*_*p *_= 0.89 for the classification, but 0.26 for a random distribution. Figure 3 shows how these values depend on the number of classes considered. Most of the information is given by about the 50 most important classes. By eye, the details are different to Figure 2, but the trend is clear. A classification may have 300 classes, but some tens of classes explain the bulk of the data. The rest of the classes reflect less frequent protein motifs. Numerically, the classification behaves more like one with only tens of classes. The small prior weights (*w*_*i *_in eq. 2) mean that these classes rarely come into play.

Given these overall properties, one can consider some example results from each type of classification.

### Sequence classification and alignment

This type of classification is included as a matter of principle, rather than practical use. There are, however, two reasons why it may have been of interest. Firstly, if one believes in the importance of sequence motifs, this could be a method for finding them. Practically all motif finding methods use some form of supervised learning (training from known data) [59-61]. The approach in this classification is simply to look for statistically significant patterns without any knowledge of function. Secondly, one might hope that patterns of amino acid probabilities are a sequence signal which would be preserved over longer evolutionary time-scales than simple sequence similarity. In this case, one could align protein sequences using the similarity based on eq 4.

First we consider whether there are some statistical patterns which are so strong and distinct that they will be found by this kind of unsupervised learning/classification. The answer is yes, but it is of no practical use. For fragments of length *k *= 6, the most statistically unusual class, as measured by the cross entropy, is HHHHHH. The second most unusual class was another common sequence tag. The other classes may be interpreted in terms of chemical properties, but it is more sensible to refrain from over-interpretation. This kind of unsupervised learning is not the best way to recognise biologically interesting sequence motifs.

Next, we briefly consider the question of sequence alignment using a score matrix based on similarities of class probability vectors (eq. 4). With the set of 2 902 distantly related protein pairs, alignments were calculated, models constructed and the alignment quality measured as described under Methods. For comparison, the same procedure was done with conventional pair-wise alignments based on a blosum62 substitution matrix [2]. The same optimization of gap penalties and matrix zero level was then calculated after filling score/alignment matrices with gaussian distributed random numbers. Figure 1 compares the results from the different approaches. The cost function (eq. 6) is based on the similarity of distance matrices (eq. 5), so even with random elements in the score matrix, the score will not be zero due to small fragments of similar structure. On this set of remote homologues, the more expensive method does not produce better alignments than those using a conventional substitution matrix. Although it is technically interesting to find a genuinely new scoring scheme for sequence alignments, it is more useful to consider this methodology in a context where it seems to be very effective.

### Structure alignment

Unlike a pure sequence-based classification, the pure structure-based classification leads to a directly useful application (structural alignment) and often easily interpretable results. We concentrate on results from a classification with fragment length *k *= 6 and 248 classes. Not surprisingly, the three most populated classes are recognisable classic secondary structure, but soon one reaches classes which may or may not have literature names. The practical application of this classification is more interesting than a reinvestigation of protein structural motifs. When the vectors in eq. 4 are based only on structural properties, they form the basis of a swift and robust protein structure alignment method, available as a web service [62] and fast enough to search a set of 17 000 representative protein folds in minutes.

Firstly, one can look at the very gross average behaviour and compare the quality of the alignments with those from the same methodology using sequences or conventional sequence alignment (previous section). Figure 1 shows the value of the testing function from the optimization described above (2 902 remote protein pairs) and the bar labelled "struct frag" refers to the structure-based alignment with this methodology. As expected, when aligning pairs of proteins with weak sequence homology, a structure based method performs much better. One may also note that the bars never go below -0.7. This reflects the fact that one is working with protein pairs whose structures are often somewhat dissimilar and the function only approaches -1 as structures become identical.

One can look at average performance, but when comparing protein alignments, it may be that there are many methods which perform similarly, even with approaches based on methods ranging from local distance information mean field methods [6-35,63-66]. In this case, it is more important to look for examples which characterise a method and where the results reflect the peculiarities of a technique.

**Figure 4:**
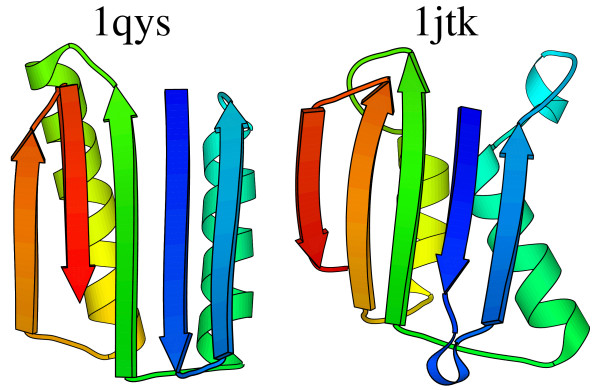
**Putative homologue of 1qys**. Aligned residues from 1qys and 1jtk superimposed. Aligned residues have the same colour. Drawn with molscript [83].

To make the point, we consider two extreme examples, one of which may suggest a weakness in the implementation here. The first example is 1qys. This was deliberately constructed so as to have a unique topology [67]. By design, it should have no structural homologues. Searches with two example reputable servers [23,44] find some similar structures with alignments of 71 or less residues. Searching with this methodology finds the same hits, but also an interesting candidate similarity ranked fourth in its list. Figure 4 shows the 90 aligned residues from 1qys to 1jtk. The colour coding is such that aligned residues in the two structures have the same colour. The potential problem here is clear. The two left-most (C-terminal) β-strands in protein 1jtk are oriented the wrong way. This alignment only requires a gap of length two. A partisan could argue that this is a significant similarity. One could also argue that since the arrangement of a β-strand is wrong, the result should be thrown away.

**Figure 5:**
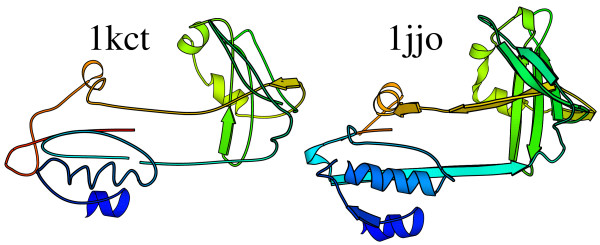
**Structural alignment of 1kct and 1jjo**. Aligned residues as calculated by the classification probability vector method are shown. Of the 374 residues present in the 1kct coordinates, 186 are aligned to sites in 1jjo chain C with 20 % sequence identity. Aligned residues have the same colour.

Secondly one can consider a protein with little regular secondary structure. The protein data bank was searched for a chain with more than 100 residues, whose structure was determined by X-ray crystallography and with less than 7 % annotated α-helix or β-strand. The first protein found was 1kct [68], an α-1-antitrypsin at 3.5 Å resolution, partially shown on the left of Figure 5. Many homologues of this protein can be found by a simple sequence search, but this structure seems to be so distorted, that a method based on recognising regular secondary structure finds no similar structures in the protein data bank [23]. Even a method based on distance matrix similarities does not find any significant structural homologues [8]. Scanning the protein data bank with 1kct and this probabilistic methodology yields a series of anti-trypsins, the most remote of which (14^th ^rank) is 1jjo shown on the right hand side of the figure. The similarity by eye is clear, but the irregularity in the query structure renders it a difficult case for some programs. Obviously, one example does not mean the code described here is in any sense better than other structure similarity finding programs. It is a deliberately chosen extreme example which highlights different properties of this methodology.

### Combined sequence and structure alignment

**Figure 6:**
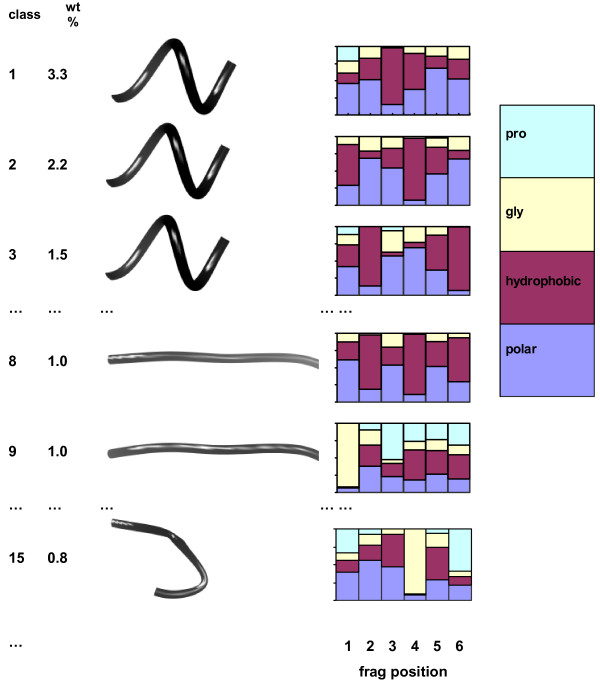
**Fragment classes from combined sequence and structure**. Class is the class number, ranked by weight, wt: the statistical weight of the class. Bar plots show the prevalence of residue types at each position, where hydrophobic is the set of residues: l, i, m, v, f, y, c, w and polar is d, e, n, k, s, q, t, r, h, a.

We have considered alignments based on class probability vectors where the original descriptors came from protein sequence (Bernoulli distributions) or structure (Gaussian distributions). The methodology implied by eq. 2 and 3 can also be applied to the mixture model including both sequence and structure information. This means one can calculate true combined sequence and structure alignments. Firstly, it is easy to see why this approach will differ from either a pure sequence or structure method. To make the point, Figure 6 shows some classes from a classification with fragment length *k *= 6 and 267 classes. The structural fragments were constructed using the *φ *and *ψ *angles from each of the 6 bivariate Gaussian distributions in the class. The residue probabilities in the bar plots are scaled relative to background probabilities, so a 1/4 high bar at a position in a fragment would mean that the probability of an amino acid type simply follows the background distribution.

The three classes with the highest statistical weight are structurally indistinguishable α-helical, but differ in their sequence profile. The second and third classes show the periodicity of amphipathic helices. Two example β-strand classes are shown which again differ in their sequence propensities. The last example (class 15) at the bottom of the plot shows a different property. The amino acid probabilities do not differ too much from background probabilities, except at position 4, which almost has to be a glycine. Looking at the fragment, it is clear that this is part of a classic, well characterised turn [69,70].

**Figure 7:**
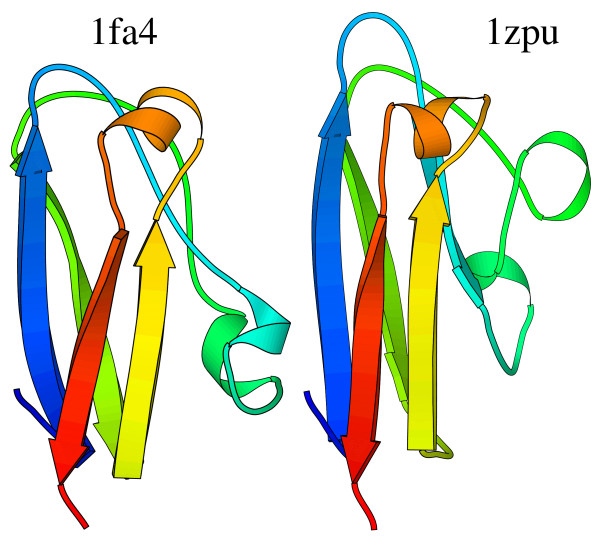
**Simultaneous sequence and structure alignment of 1fa4 and 1zpu**. Residues 22 to 105 from 1fa4 aligned to residues 58–145 of 1zpu (chain A). Aligned residues have the same colour.

These fragments from the combined mixture model were also used for alignments and the gross performance is given by the bar in Figure 1 labelled "seq+struct frag". Averaging over the 5 804 models, there seems to be little difference between the combined method and pure structural alignments. Of course, there are many differences in individual pairs, especially where similarity is very weak. As an example, we searched for a case which is slightly counter to what one would expect. Usually, one expects protein sequences to diverge faster than structure and this is the basis for discussions on surprising protein similarities [11]. Given the methodology available here, we looked for an example in the other direction – a pair of proteins where the structural similarity score is poor, but an alignment using sequence and structure descriptors scored well. An example combined alignment is shown in Figure 7. No pure structural alignment is shown. Using either 1fa4 or 1zpu (chain A) as a search model, neither 1zpu or 1fa4 was found as a structural homologue with four servers [11,14,23,42-44,71]. A pure structure-based search using our code found no significant hit and explicitly calculating an alignment aligned 90 residues (2 single gaps) but with a root mean square difference calculated at C^*α *^atoms of 16.5 Å.

A combined sequence to structure alignment resulted in the superposition of Figure 7. Within the 89 aligned residues there is only 13 % sequence identity and it only covers about 17 % of the 529 residues from chain A of 1zpu. It would be reasonable to doubt its significance. In fact, both are copper containing proteins involved in redox chemistry, albeit one from algae [72] the other from yeast [73]. Interestingly, it is possible to find a very remote sequence connection between the two proteins. Using the sequence of 1fa4 as a query, a sequence profile was built using the non-redundant protein sequence database and used to search against protein data bank sequences [58]. This finds 1zpu as a potential homologue with a very poor *e*-value (0.02). By itself, this would also not be considered significant. Most persuasively, the iterated sequence search from psi-blast aligns residues 34 to 123 of 1zpu and the combined sequence/structure alignment using our code aligns almost exactly the same stretch (residues 37 to 124). This appears to be simply an example of normal divergent evolution, but it is an example of where structure has diverged to the point where a simple structural superposition is not conclusive.

Again one should be clear that this kind of result is not in its own significant. When one is dealing with remote homologues, different programs will produce different results. With enough time, one would be able to find alignments which are found with other codes, but missed or miscalculated using our methods. The interesting point is that there is one method and one scoring scheme which can operate on both protein sequences and structures.

## Discussion

Clearly, it is possible to have a single probabilistic methodology for finding similarities based on sequence, structure or simultaneous sequence and structure. The question is whether one would want to. The application to sequence alignment is interesting, but not obviously useful. The pure structure alignment, based on continuous descriptors is obviously useful and available as a web service [62]. The combination of sequence and structure descriptors is an unexploited method which has different properties to other alignment techniques which leads to two future possibilities. Firstly, it is accepted dogma that protein sequences evolve faster than structure, so one can detect similarities even when sequence homology is not significant [74]. With the tools here, it is relatively easy to search for cases where structural alignment is weak, but combined alignment appears to be significant. Secondly, there is the general question of remote homology detection. Protein structure searches are an essential tool when proteins have diverged so much that sequence similarity is, by itself, not significant. The question is whether combining all available descriptors will usually yield even better results. Although we give one example above (1fa4 and 1zpu) it needs more testing and the collection of new benchmarks to find if it is useful and if so, in what regime of similarity. From one point of view, one should use all available information (sequence as well as structure). From another point of view, this may not be true. Sequence mutations are often modelled as random events or walks through possible sequences [75,76]. If two sequences have diverged such that there is little sequence similarity, adding sequence information will introduce noise as well as signal.

The methodology is in most senses rather unusual and there are some assumptions and limitations. If one feels the underlying statistical models are a good representation of protein data, then the rest of the procedure is completely justified. Of course the underlying models are not perfect. Gaussian distributions are mainly chosen for convenience and one knows that there are some correlations which could be included. The distributions in this work accounted for *φ*, *ψ *correlations within a residue, but test calculations on smaller data sets suggest that in a small number of classes there are correlations between neighbouring residues which could be accounted for. The problem is that there are currently 18 parameters per class in a pure structure classification with *k *= 6. Using a full covariance matrix results in 27 parameters per class.

There are already many protein fragment classifications in the literature, but usually with a different philosophy. Generally, these use a structure classification and then see which sequence patterns fit to each structure motif (or *vice versa*). They also require some similarity measure between clusters [35-38,54,77-81]. The methodology here is based on a mixture model which can treat all these properties simultaneously and this leads to a very different kind of result. As shown in Figure 6, a single structural motif can accommodate different sequence patterns. These are detected in this work since all the descriptors are considered simultaneously. Figure 6 shows half a dozen classes, but if one were to look through the other 261 classes, there are numerous examples of different sequence patterns fitting to a basic structural unit.

This raises the question as to which is most important when sequence and structure are combined. Unfortunately, there is no simple answer since it varies from class to class and site to site. As shown in Figure 6, a class may have relatively flat distributions for amino acids or sometimes a particular site has a distribution far from background probability. In crude terms, summing over all observations and all classes, the structural descriptors are about 3 1/2 times more important than the sequence descriptors in terms of discriminating.

The next major difference compared to other classification schemes is the application. Usually this is connected to prediction. If one has a sequence clustering one can collect structure properties to make structural predictions or *vice versa*. The Bayesian classification scheme used in this work has been used for this purpose [47], but not in this work. Here, we are interested in a single kind of similarity measure which operates in different contexts.

The results (or the lack thereof) for pure sequence alignment make it clear that this methodology will not displace conventional sequence-based methods. The results for structure and combined sequence and structure are far more promising. There are several reasons. Firstly, there are no preconceptions of regular protein structure. If some motif is statistically described it is part of the model. There is little preference for strands, helices or recognized turns over other motifs. Next, the method handles unusual structures. When faced with a fragment which has never been seen before, it will not be placed into any particular class. It will have some probability of being in a few classes. Any similar fragment, even if it has never been observed before, will have a similar set of class membership probabilities and will be recognized as similar. Next, the procedure is rather free of thresholds. The probability of similarity (eq. 4) runs smoothly between 0 and 1. There are no absolute matching steps necessary. Finally, each residue in a protein is involved in 2*k *- 1 overlapping fragments. In this work, this means that each element in a similarity matrix reflects the properties of 11 residues. These factors are probably why the method is rather tolerant of poor structures such as the example in Figure 5.

The procedure is also rather swift. To make database searches fast, class probability vectors for representative chains can be precalculated. This leaves the normal quadratic running time for the dynamic programming step. Compared to simple sequence alignment, this is slower by a constant factor since the normal table lookup from a substitution matrix is replaced by a dot product calculation.

The approach may be useful for pure structure alignment, but its performance needs to be demonstrated quantitatively in terms of accuracy and speed, rather than by the proof-of-concept examples given here. The main advance is that one can mix sequence and structure on equal probabilistic terms without any *ad hoc *weighting of the terms. Since the methodology is fast enough for phylogenetic calculations, we are now interested in finding examples where the different approach yield different results. It remains to be seen which is more reliable or at least persuasively believable.

## Conclusion

With modest assumptions, it is possible to combine protein sequence and structure in one framework for protein alignment and comparison. Larger scale testing needs to be done to estimate its significance. The server is available for structure comparisons [62] and all software is free for download [82].

## Competing interests

The author(s) declare that they have no competing interests.

## Authors' contributions

All authors have read and approved the manuscript and contributed equally to this work.

## References

[B1] Dayhoff M, Schwartz R, Orcutt B, Dayhoff M (1978). A model of evolutionary change in proteins. Atlas of Protein Sequence and Structure.

[B2] Henikoff S, Henikoff JG (1992). Amino acid substitution matrices from protein blocks. Proc Natl Acad Sci USA.

[B3] Jung J, Lee B (2000). Use of residue pairs in protein sequence-sequence and sequence-structure alignments. Protein Sci.

[B4] Gonnet GH, Cohen MA, Benner SA (1994). Analysis of Amino-Acid Substitution During Divergent Evolution – the 400 by 400 Dipeptide Substitution Matrix. Biochem Biophys Res Commun.

[B5] Crooks GE, Green RE, Brenner SE (2005). Pairwise alignment incorporating dipeptide covariation. Bioinformatics.

[B6] Zuker M, Somorjai RL (1989). The alignment of protein structures in three dimensions. Bull Math Biol.

[B7] Russell RB, Barton GJ (1992). Multiple protein sequence alignment from tertiary structure comparison. Proteins.

[B8] Holm L, Sander C (1993). Protein-Structure Comparison by Alignment of Distance Matrices. J Mol Biol.

[B9] Subbiah S, Laurents DV, Levitt M (1993). Structural similarity of DNA-binding domains of bacteriophage repressors and the globin core. Curr Biol.

[B10] Alexandrov NN (1996). SARFing the PDB. Protein Eng.

[B11] Gibrat J-F, Madej T, Bryant SH (1996). Surprising similarities in structure comparison. Curr Opin Struct Biol.

[B12] Orengo CA, Taylor WR (1996). SSAP: Sequential structure alignment program for protein structure comparison. Method Enzymol.

[B13] Suyama M, Matsuo Y, Nishikawa K (1997). Comparison of protein structures using 3D profile alignment. J Mol Evol.

[B14] Shindyalov IN, Bourne PE (1998). Protein structure alignment by incremental combinatorial extension (CE) of the optimal path. Protein Eng.

[B15] Holm L, Park J (2000). DaliLite workbench for protein structure comparison. Bioinformatics.

[B16] Jung J, Lee B (2000). Protein structure alignment using environmental profiles. Protein Eng.

[B17] Lackner P, Koppensteiner WA, Sippl MJ, Domingues FS (2000). ProSup: a refined tool for protein structure alignment. Protein Eng.

[B18] Ortiz AR, Strauss CEM, Olmea O (2002). MAMMOTH (Matching molecular models obtained from theory): An automated method for model comparison. Protein Sci.

[B19] Shatsky M, Nussinov R, Wolfson HJ (2002). Flexible protein alignment and hinge detection. Proteins.

[B20] Blankenbecler R, Ohlsson M, Peterson C, Ringnér M (2003). Matching protein structures with fuzzy alignments. Proc Natl Acad Sci USA.

[B21] Kawabata T (2003). MATRAS: a program for protein 3D structure comparison. Nucl Acids Res.

[B22] Ilyin VA, Abyzov A, Leslin CM (2004). Structural alignment of proteins by a novel TOPOFIT method, as a superimposition of common volumes at a topomax point. Protein Sci.

[B23] Krissinel E, Henrick K (2004). Secondary-structure matching (SSM), a new tool for fast protein structure alignment in three dimensions. Acta Cryst D.

[B24] Ochagavia ME, Wodak H (2004). Progressive combinatorial algorithm for multiple structural alignments: Application to distantly related proteins. Proteins.

[B25] Shapiro J, Brutlag D (2004). FoldMiner and LOCK 2: protein structure comparison and motif discovery on the web. Nucl Acids Res.

[B26] Carpentier M, Brouillet S, Pothier J (2005). YAKUSA: A fast structural database scanning method. Proteins.

[B27] Chen L, Zhou T, Tang Y (2005). Protein structure alignment by deterministic annealing. Bioinformatics.

[B28] Chen Y, Crippen GM (2005). A novel approach to structural alignment using realistic structural and environmental information. Protein Sci.

[B29] Zhang Y, Skolnick J (2005). TM-align: a protein structure alignment algorithm based on the TM-score. Nucl Acids Res.

[B30] Zhu JH, Weng ZP (2005). FAST: A novel protein structure alignment algorithm. Proteins.

[B31] Konagurthu AS, Whisstock JC, Stuckey PJ, Lesk AM (2006). MUSTANG: A multiple structural alignment algorithm. Proteins.

[B32] Lisewski AM, Lichtarge O (2006). Rapid detection of similarity in protein structure and function through contact metric distances. Nucl Acids Res.

[B33] Taubig H, Buchner A, Griebsch J (2006). PAST: fast structure-based searching in the PDB. Nucl Acids Res.

[B34] Oldfield TJ (2007). CAALIGN: a program for pairwise and multiple protein-structure alignment. Acta Cryst D.

[B35] Friedberg I, Harder T, Kolodny R, Sitbon E, Li ZW, Godzik A (2007). Using an alignment of fragment strings for comparing protein structures. Bioinformatics.

[B36] Camproux AC, Tuffery P, Chevrolat JP, Boisvieux JF, Hazout S (1999). Hidden Markov model approach for identifying the modular framework of the protein backbone. Protein Eng.

[B37] Hunter CG, Subramaniam S (2003). Protein fragment clustering and canonical local shapes. Proteins.

[B38] Sander O, Sommer I, Lengauer T (2006). Local protein structure prediction using discriminative models. BMC Bioinformatics.

[B39] Simons KT, Ruczinski I, Kooperberg C, Fox BA, Bystroff C, Baker D (1999). Improved recognition of native-like protein structures using a combination of sequence-dependent and sequence-independent features of proteins. Proteins.

[B40] Berman HM, Westbrook J, Feng Z, Gilliland G, Bhat TN, Weissig H, Shindyalov IN, Bourne PE (2000). The Protein Data Bank. Nucl Acids Res.

[B41] Li WZ, Jaroszewski L, Godzik A (2001). Clustering of highly homologous sequences to reduce the size of large protein databases. Bioinformatics.

[B42] Holm L, Sander C (1994). The FSSP database of structurally aligned protein fold families. Nucl Acids Res.

[B43] Holm L, Sander C (1996). The FSSP database: Fold classification based on structure structure alignment of proteins. Nucl Acids Res.

[B44] Holm L, Sander C (1997). Dali/FSSP classification of three-dimensional protein folds. Nucl Acids Res.

[B45] Holm L, Sander C (1998). Touring protein fold space with dali/FSSP. Nucl Acids Res.

[B46] Dempster AP, Laird NM, Rubin DB (1977). Maximum likelihood from incomplete data via the EM algorithm. J R Stat Soc B.

[B47] Wurst server. http://www.zbh.uni-hamburg.de/wurst.

[B48] Cheeseman P, Stutz J, Fayyad U, Piatetsky-Shapiro G, Smyth P, Uthurusamy R (1995). Bayesian Classification (Autoclass): Theory and Results. Advances in Knowledge Discovery and Data Mining.

[B49] Gotoh O (1982). An improved algorithm for matching biological sequences. J Mol Biol.

[B50] Smith TF, Waterman MS (1981). Identification of Common Molecular Subsequences. J Mol Biol.

[B51] Onuchic JN, Luthey-Schulten Z, Wolynes PG (1997). Theory of protein folding: The energy landscape perspective. Annu Rev Phys Chem.

[B52] Goldstein RA, Luthey-Schulten ZA, Wolynes PG (1992). Protein tertiary structure recognition using optimized Hamiltonians with local interactions. Proc Natl Acad Sci USA.

[B53] Levitt M (1983). Molecular dynamics of native protein. II. Analysis and nature of motion. J Mol Biol.

[B54] Rooman MJ, Rodriguez J, Wodak SJ (1990). Automatic definition of recurrent local structure motifs in proteins. J Mol Biol.

[B55] Crippen GM (1996). Easily searched protein folding potentials. J Mol Biol.

[B56] Havel TF (1990). The sampling properties of some distance geometry algorithms applied to unconstrained polypeptide chains: a study of 1830 independently computed conformations. Biopolymers.

[B57] Russell A, Torda AE (2002). Protein sequence threading – averaging over structures. Proteins.

[B58] Altschul SF, Madden TL, Schaffer AA, Zhang J, Zhang Z, Miller W, Lipman DJ (1997). Gapped BLAST and PSI-BLAST: a new generation of protein database search programs. Nucl Acids Res.

[B59] Attwood TK, Blythe MJ, Flower DR, Gaulton A, Mabey JE, Maudling N, McGregor L, Mitchell AL, Moulton G, Paine K, Scordis P (2002). PRINTS and PRINTS-S shed light on protein ancestry. Nucl Acids Res.

[B60] Liu J, Rost B (2003). Domains, motifs and clusters in the protein universe. Curr Opin Chem Biol.

[B61] Sigrist CJA, Cerutti L, Hulo N, Gattiker A, Falquet L, Pagni M, Bairoch A, Bucher P (2002). PROSITE: A documented database using patterns and profiles as motif descriptors. Brief Bioinform.

[B62] Salami: Protein structure similarity searches based on classification probability vectors. http://www.zbh.uni-hamburg.de/salami.

[B63] Guyon F, Camproux AC, Hochez J, Tuffery P (2004). SA-Search: a web tool for protein structure mining based on a Structural Alphabet. Nucl Acids Res.

[B64] Koehl P (2001). Protein structure similarities. Curr Opin Struct Biol.

[B65] Levine M, Stuart D, Williams J (1984). A Method for the Systematic Comparison of the 3-Dimensional Structures of Proteins and Some Results. Acta Cryst A.

[B66] Martinez L, Andreani R, Martinez JM (2007). Convergent algorithms for protein structural alignment. BMC Bioinformatics.

[B67] Kuhlman B, Dantas G, Ireton GC, Varani G, Stoddard BL, Baker D (2003). Design of a novel globular protein fold with atomic level accuracy. Science.

[B68] Song HK, Lee KN, Kwon KS, Yu MH, Suh SW (1995). Crystal structure of an uncleaved alpha 1-antitrypsin reveals the conformation of its inhibitory reactive loop. FEBS Lett.

[B69] Wilmot CM, Thornton JM (1988). Analysis and Prediction of the Different Types of β-Turn in Proteins. J Mol Biol.

[B70] Wilmot CM, Thornton JM (1990). β-Turns and their distortions: a proposed new nomenclature. Protein Eng.

[B71] Shindyalov IN, Bourne PE (2001). A database and tools for 3-D protein structure comparison and alignment using the Combinatorial Extension (CE) algorithm. Nucl Acids Res.

[B72] Ma L, Jorgensen AMM, Sorensen GO, Ulstrup J, Led JJ (2000). Elucidation of the Paramagnetic *R *_1 _Relaxation of Heteronuclei and Protons in Cu(II) Plastocyanin from Anabaena variabilis. J Am Chem Soc.

[B73] Taylor AB, Stoj CS, Ziegler L, Kosman DJ, Hart PJ (2005). The copper-iron connection in biology: Structure of the metallo-oxidase Fet3p. Proc Natl Acad Sci USA.

[B74] Holm L, Sander C (1996). Mapping the protein universe. Science.

[B75] Ewens WJ, Grant GR (2001). Statistical methods in bioinformatics.

[B76] Jukes TH, Cantor CR, Munro HN (1969). Evolution of protein molecules. Mammalian protein metabolism.

[B77] Camproux AC, Gautier R, Tuffery P (2004). A hidden Markov model derived structural alphabet for proteins. J Mol Biol.

[B78] Camproux AC, Tuffery P, Buffat L, Andre C, Boisvieux JF, Hazout S (1999). Analyzing patterns between regular secondary structures using short structural building blocks defined by a hidden Markov model. Theor Chem Account.

[B79] Dong QW, Wang XL, Lin L (2007). Methods for optimizing the structure alphabet sequences of proteins. Comput Biol Med.

[B80] Simons KT, Kooperberg C, Huang E, Baker D (1997). Assembly of protein tertiary structures from fragments with similar local sequences using simulated annealing and Bayesian scoring functions. J Mol Biol.

[B81] Tendulkar AV, Ogunnaike B, Wangikar PP (2007). Protein local conformations arise from a mixture of Gaussian distributions. J Biosci.

[B82] Wurst. http://backpan.perl.org/authors/id/W/WU/WURST/.

[B83] Kraulis PJ (1991). MOLSCRIPT: a program to produce both detailed and schematic plots of protein structures. J Appl Crystallogr.

